# Micro-CT, Mechanical, and Histological Examination of the Effect of Local Adjuvants on Porcine Cortical Bone Following Intralesional Curettage of Bone Tumors

**DOI:** 10.1245/s10434-024-15397-4

**Published:** 2024-05-14

**Authors:** Vasileios Apostolopoulos, Petr Boháč, Petr Marcián, Iva Staniczkova Zambo, Lukáš Pazourek, Michal Mahdal, Jakub Neradil, Tomáš Návrat, Tomáš Tomáš

**Affiliations:** 1 First Department of Orthopaedic Surgery, St. Anne’s University Hospital and Faculty of Medicine, Masaryk University, Brno, Czech Republic; 2 Institute of Solid Mechanics, Mechatronics and Biomechanics, Faculty of Mechanical Engineering, University of Technology, Brno, Czech Republic; 3 First Department of Pathology, St. Anne’s University Hospital and Faculty of Medicine, Masaryk University, Brno, Czech Republic; 4 Laboratory of Tumor Biology, Department of Experimental Biology, Faculty of Science, Masaryk University, Brno, Czech Republic; 5 International Clinical Research Center, St. Anne’s University Hospital, Brno, Czech Republic

**Keywords:** Local adjuvants, Giant cell tumor of bone, Micro-CT, Bone hardness, Bone necrosis

## Abstract

**Background and Objectives:**

Curettage is the removal of a tumor from the bone while preserving the surrounding healthy cortical bone, and is associated with higher rates of local recurrence. To lower these rates, curettage should be combined with local adjuvants, although their use is associated with damage to nearby healthy bone.

**Objective:**

The purpose of this analysis is to determine the effect of local adjuvants on cortical porcine bone by using micro-computed tomography (micro-CT) along with histological and mechanical examination.

**Methods:**

Local adjuvants were applied to porcine specimens under defined conditions. To assess changes in bone mineral density (BMD), a micro-CT scan was used. The pixel gray values of the volume of interest (VOI) were evaluated per specimen and converted to BMD values. The Vickers hardness test was employed to assess bone hardness (HV). The depth of necrosis was measured histologically using hematoxylin and eosin-stained tissue sections.

**Results:**

A noticeable change in BMD was observed on the argon beam coagulation (ABC) sample. Comparable hardness values were measured on samples following electrocautery and ABC, and lowering of bone hardness was obtained in the case of liquid nitrogen. Extensive induced depth of necrosis was registered in the specimen treated with liquid nitrogen.

**Conclusion:**

This study determined the effect of local adjuvants on cortical bone by using micro-CT along with histological and mechanical examination. Phenolization and liquid nitrogen application caused a decrease in bone hardness. The bone density was affected in the range of single-digit percentage values. Liquid nitrogen induced extensive depth of necrosis with a wide variance of values.

Locally aggressive tumors, benign or low-grade malignant tumors confined to a single area of the bone, are commonly treated by intralesional curettage.^1–3^ Removal of the tumor from the bone cavity while sparing the surrounding healthy cortical bone preserves the function of the adjacent joint and is associated with less damage to the overlying soft tissues compared with en bloc resection.^4–6^ Intralesional curettage is a treatment method used for various bone tumors, including giant cell tumors of bone, chondroblastomas, aneurysmal bone cysts and atypical cartilaginous tumors, and serves as a palliative approach for metastatic disease.^7,8^ Many orthopedic departments achieved better clinical outcomes with intralesional curettage than with tumor bone complete resection.^9–12^ Particularly in cases without extraosseous infiltration, curettage is the surgical treatment of choice.^4,10–13^

The irregular shape of the tumor cavity of some types of tumors makes it difficult to remove microscopic and even macroscopic tumor residues, and as a result, higher rates of local recurrence after curettage of the tumor have been recorded.^4,14,15^ To lower those rates, curettage should be combined with other local treatments to help prevent the tumor from recurring.^4,6,16^ The use of local adjuvants has been documented to decrease recurrence rates by 10–30%, with effectiveness varying based on tumor type and the specific local adjuvant employed.^8,17–19^ An addition to curettage may be high-speed burring.^20^ The goal of adjuvant therapy is to eliminate any remaining viable tumor cells and achieve an adequate margin.^6,20^

Local adjuvants are chemical or physical agents applied locally. Chemical adjuvants such as phenol, ethanol, and hydrogen peroxide have been used and are applied directly after the curettage, and eventually high-speed burring, to the bone cavity at the site of the tumor.^21,22^ Specifically, phenol is a clear, toxic, alcohol-soluble liquid that causes rapid cell death through prolonged protein coagulation.^23^ Physical local adjuvants are divided into thermal ablation and cryoablation agents. Thermal ablation utilizes heat to destroy any remaining cancer cells in the affected bone.^24^ The main representatives of thermal ablation are argon beam coagulation (ABC) and electrocautery.^25^ In ABC, a beam of ionized argon gas is passed through a high-frequency alternating current to create an electrical spark that coagulates and reduces the underlying tissue.^6,25^ Electrocautery refers to a process in which a direct or alternating current is passed through a resistant metal wire electrode, generating heat that causes hemostasis or varying tissue destruction.^26^ By contrast, cryoablation uses cold, by injecting liquid nitrogen into the desired cavity or by applying nitrogen gas through a so-called ‘nitrogen spray gun’.^27^ The surgical techniques of the use of local adjuvants have been described in detail in the literature.^4,6^

Although local adjuvants are considered a beneficial tool in the prevention of tumor recurrence, their use is associated with damage to nearby healthy bone or even to surrounding soft tissue and neurovascular structures, particularly when high doses are used.^28^ Extensive bone tissue necrosis around the tumoral cavity, especially when compact bone is involved, could lead to pathological fractures.^27,29^ However, in most cases, the benefits of using adjuvants to reduce the risk of the tumor recurring or spreading outweigh the potential risks to the surrounding healthy bone.^12,15^

The purpose of this multidisciplinary analysis was to determine the effect of these chemical and thermal local adjuvants on cortical bone by using micro-computed tomography (micro-CT) along with histological and mechanical examination. Therefore, we will examine (1) the change in bone density after the application of local adjuvants; (2) the change in cortical bone hardness after the application of local adjuvants; and (3) the depth of necrosis after the application of local adjuvants.

## Materials and Methods

### Specimen Preparation and Treatment

To assess the effect of local adjuvants on cortical bone, fresh adult pig femurs were obtained from a certified slaughterhouse. All femur samples, including reference samples, were harvested from the right side of male pigs of the same age and weight. Specimens were derived from the mid-section of the diaphysis, and handled and stored identically to minimize potential sources of bias. All specimens underwent the same thorough cleaning process and were subsequently stored for up to 1 week in a freezer at − 19 °C.

A total of 35 samples were prepared, 7 for each type of local adjuvant, including reference samples. Samples were positioned into a piece of porcine muscle for better conductivity. Local adjuvants were applied to specimens under defined conditions:

1. *Argon beam coagulation group treatment method:* The argon beam coagulator was set at 120 W, as recommended for larger bones, and applied for exactly 30 s.^5^ After washing with saline, a second cycle of ABC was then performed, and the specimen was washed again by saline lavage.
2. *Electrocautery group treatment method:* The electrocautery was set at 100 W in spray mode and directly applied all over the cavity until the medular side of the diaphyseal surface was darkened.^6,24^ The specimen was then washed by saline lavage.
3. *Liquid nitrogen group treatment method:* Liquid nitrogen was sprayed out from the can toward the medular side of the diaphyseal specimens using a cryosurgical liquid nitrogen sprayer.^30,31^ Once the ice on the surface of the cavity thawed, a second cryoablation cycle was performed.
4. *Phenol group treatment method:* Gauze pads soaked with 80% phenol were positioned on the medular side of the diaphyseal specimens for a period of 4 min,^6,32^ and the specimen was then washed by saline lavage. Two cycles of phenolization were carried out.
5. *Reference group treatment method:* The reference samples were cleaned and stored exactly as the other samples, but no local adjuvant was applied. The samples were washed by saline lavage.

This study was conducted according to the guidelines of the Declaration of Helsinki and was approved by the appropriate Institutional Review Committee.

### Micro-Computed Tomography (CT) Examination

To assess changes in bone mineral density (BMD), a specimen per type of local adjuvant was scanned. The specimens were scanned using a micro-CT scanner (GE Phoenix v|tome|x L240, GE Sensing and Inspection Technologies GmbH, Wunstorf, Germany) with a voxel size of 14 μm. The bone density calibration was performed using a hydroxyapatite phantom (Micro-CT-HA D20, © QRM GmbH, Moehrendorf, Germany) included during scanning. This phantom caliber consists of five cylinders with different densities—0, 50, 200, 800, and 1200 mg/cm^3^. Micro-CT images of the phantom were then used to determine bone density according to a standard procedure based on a linear relationship between hydroxyapatite density and the pixel gray values of the corresponding image.^33,34^ Due to quantitative analysis, all five samples, including the caliber, were scanned during one measurement.

A pixel gray-value threshold of 10,000 is considered between hard and air/soft tissues.

**Fig. 1:**
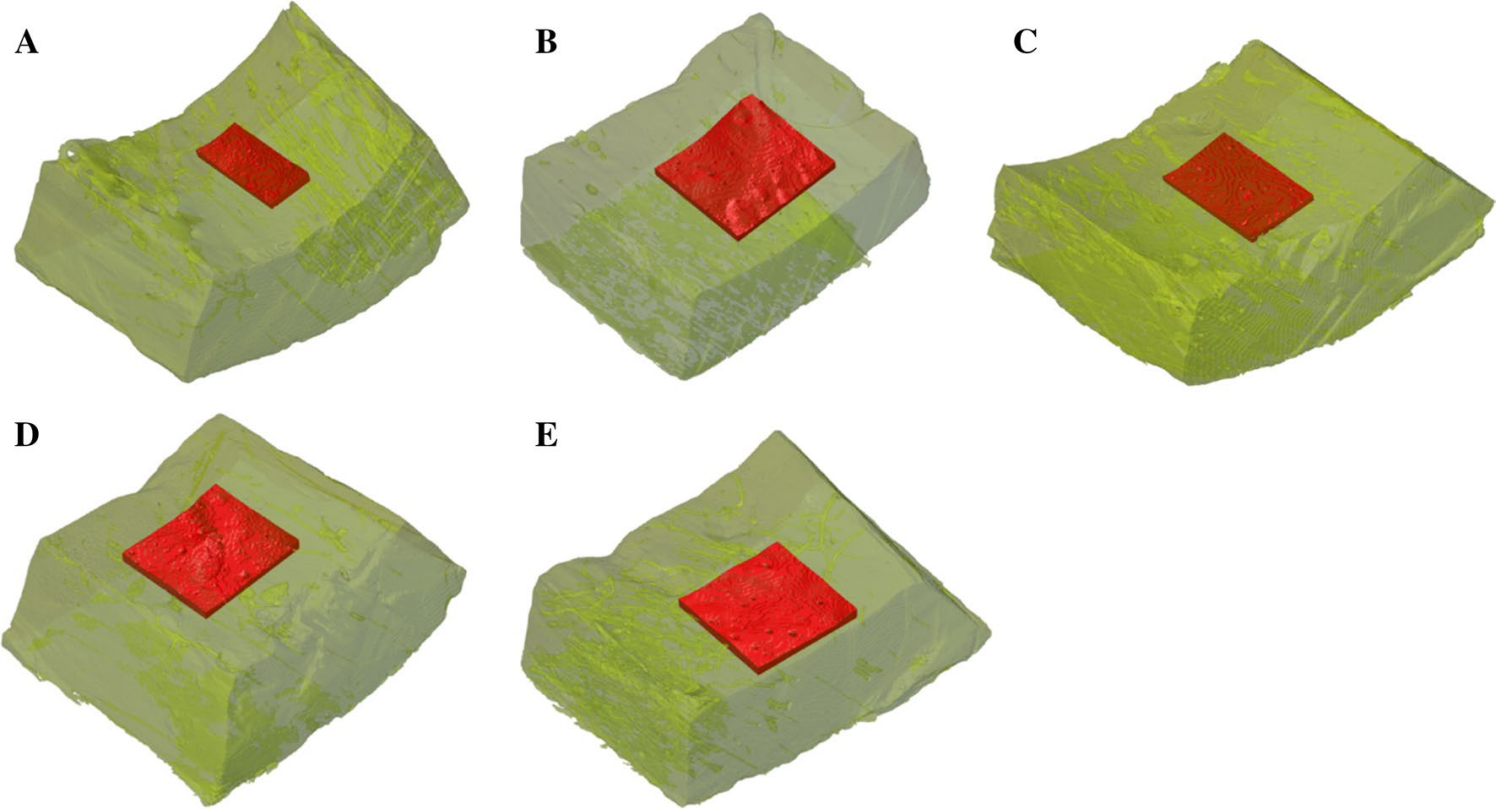
Micro-CT view of the evaluated VOI for each type of treated specimen. *Micro-CT* micro-computed tomography, *VOI* volume of interest

The pixel gray values of the volume of interest (VOI) were evaluated per specimen (Fig. 1). The representative area was on the medular surface of the diaphyseal specimen and of a similar depth (0.2–0.4 mm). The pixel gray values of the volume were recorded and converted to BMD values. The examiner was blinded to the treatment type.

### Mechanical Examination

In the assessment of induced mechanical changes, the Vickers hardness test, a commonly recognized method for measuring the hardness of a variety of materials, including cortical bone, was employed.^35^ This test uses a micro indentation with an indenter to the prepared specimen surface. The indenter has a square-based pyramid shape, and is made of a very hard material.^36^ This indenter was pressed to the medular surface of the specimen with the specified force for the indicated duration.

In this case, the applied load was 50 g for 10 s, using a Vickers hardness testing machine (INNOVATEST FALCON 800G2). The resulting indentation size is measured by high-magnification optics, and software developed for such a measurement evaluates the Vickers hardness (HV) based on the length of the two resulting diagonals of the indentation. The HV value (HV 0.05/10 s) represents the hardness of the material in the sense of resistance to deformation.^35^ Higher HV values indicate harder materials. Three specimens per type were measured, and three indentations were made for each specimen, to ensure that the impact of the potential variation would be minimized. A total of nine indentations were made for each type of adjuvant and reference sample. The mean HV value per specimen was recorded, namely three mean values per type of adjuvant. The examiner was blinded to the treatment type.

### Histological Examination

To assess the induced depth of necrosis, tissue specimens were fixed in 10% neutral buffered formalin, embedded in paraffin, and routinely processed. Due to significant ossification of cortical bone, the material had to undergo decalcification in a solution of 8% hydrochloric acid with ferric chloride at room temperature. Decalcification was completed after 18 days.^37^ Hematoxylin and eosin-stained tissue sections were evaluated using a Nikon microscope Eclipse Ci with a Nikon DS-Fi2 camera, and the depth of necrosis was measured using NIS-Elements D 4.13.04 software. The experienced pathologist was blinded to the treatment type.

### Statistical Analysis

The null hypothesis of this research is that the local application of an adjuvant does not influence the density and hardness of the bone. To test this hypothesis, statistical tests for comparing two unrelated samples were used, with one serving as the reference. The initial step involved the application of the Shapiro–Wilk statistic to assess the normality of the measured data. In cases where the data deviated significantly from a normal distribution, a non-parametric statistical test was preferred for analysis; otherwise, a parametric statistical test was applied. Based on the results of the Shapiro–Wilk test, the following tests were eventually used: a parametric two‐tailed t‐test was used to compare hardness test measurements, and a non-parametric Mann-Whitney U test was used to compare bone density values for each type of treated specimen. A significance level of 0.001 was used for all statistical tests. Statistical analysis was performed using R software (version 4.0.5) in the RStudio development environment.

## Results

### Micro-CT Evaluation of Bone Density

The mean BMD value of the reference sample was 1018.3 mg/cm^3^, measured in a VOI of 1.068 mm^3^. A wide variance of BMD values was not registered between the types of local adjuvants. Phenolization (1071.8 mg/cm^3^) and liquid nitrogen specimens had similar (1055.6 mg/cm^3^) average BMD values (Table 1). The highest mean difference from the reference sample was measured in the case of the ABC sample (924.7 mg/cm^3^) in a VOI of 2.348 mm^3^ (Fig. 1).

**Table 1:** Bone density values (mg/cm^3^) for each type of treated specimen and mean difference from the reference specimen (results of the Mann–Whitney U tests)

Local adjuvant	No. of pixels	VOI (mm^3^)	Bone density mean value (mg/cm^3^)	Mean difference	U	*p* value
Reference (A)	389,198	1.068	1018.3	–	–	–
Electrocautery (B)	532,408	1.461	1082.1	63.8	317	< 0.001
Phenol (C)	1,191,943	3.271	1071.8	53.5	1209	< 0.001
Argon beam coagulation (D)	855,520	2.348	924.7	−93.6	24,341	< 0.001
Liquid nitrogen (E)	1,132,214	3.107	1055.6	37.3	2588	< 0.001

*VOI* volume of interest

### Mechanical Examination of Bone Hardness

**Fig. 2:**
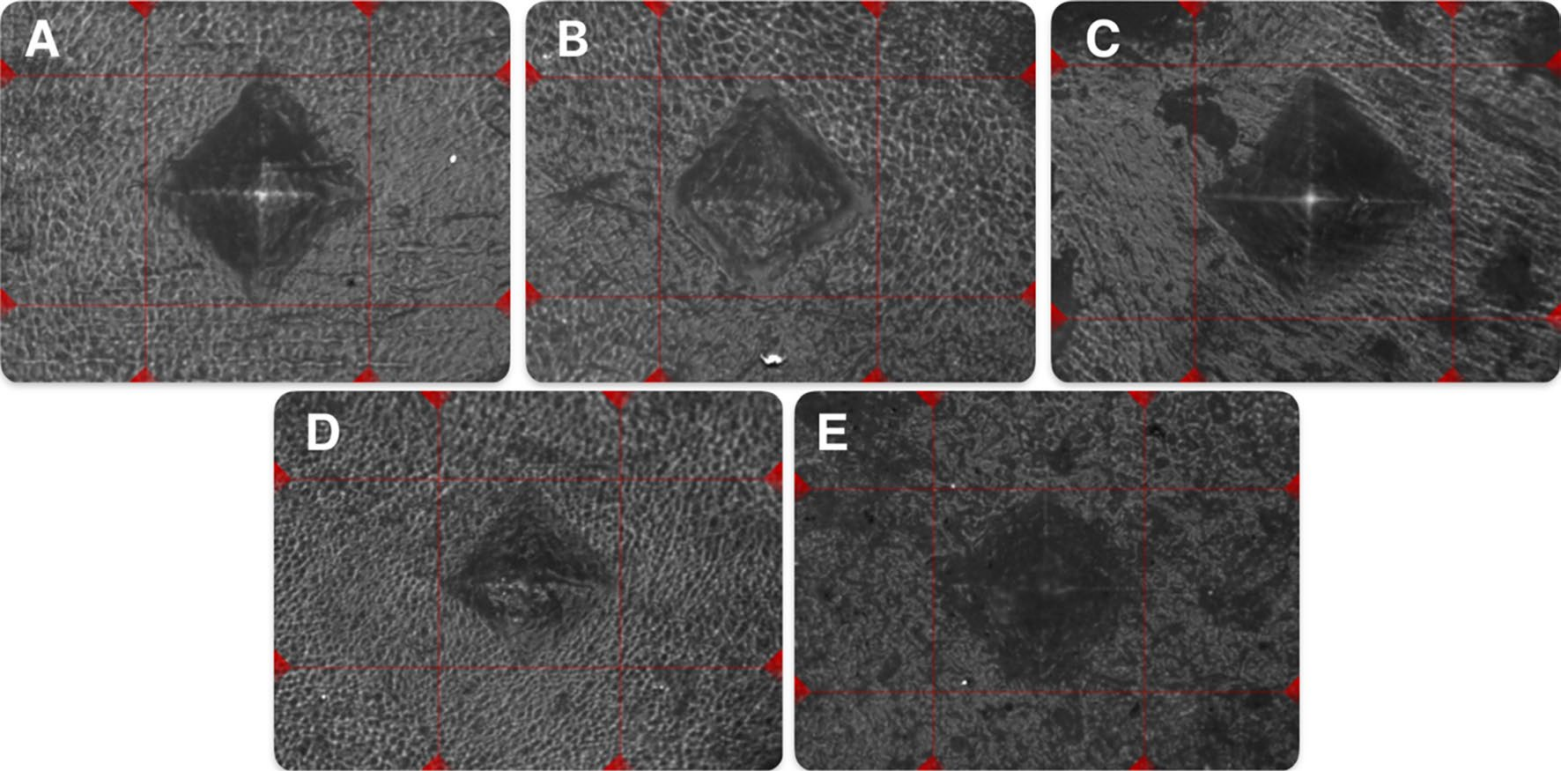
Detailed view of specimens after indentation. Types of local adjuvant specimens: **A** Reference; **B** electrocautery; **C** phenol; **D** argon beam coagulation; and **E** liquid nitrogen

**Fig. 3:**
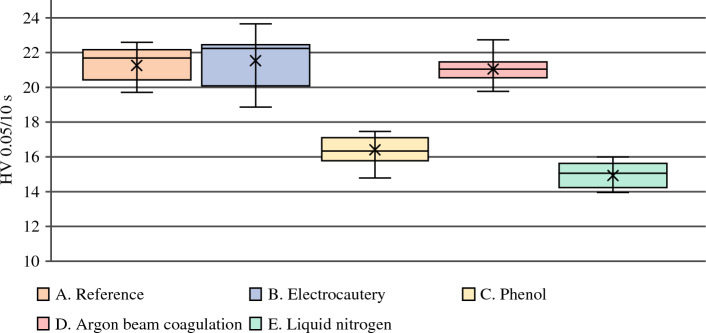
Hardness test measurement graph for each type of treated specimen with standard deviation: Types of local adjuvant specimens **A** Reference, **B** Electrocautery, **C** Phenol, **D** Argon beam coagulation and **E** Liquid nitrogen

The average HV value for the reference samples was 21.31 ± 1.11 HV 0.05/10 s. Similar HV values were measured in samples after local application of thermal adjuvants—electrocautery (mean difference − 0.310 HV 0.05/10 s, 95% confidence interval [CI] − 1.07 to 1.69; *p* = 0.640) and ABC (mean difference − 0.190 HV 0.05/10 s, 95% CI − 1.18 to 0.80; *p* = 0.690) (Table 2). By contrast, bone hardness was lowered in the case of phenolization (mean difference − 4.900 HV 0.05/10 s, 95% CI − 5.90 to − 3.89; *p* < 0.001) (Fig. 2). The local adjuvant that greatly affected the hardness of cortical bone was liquid nitrogen (mean difference: − 6.330 HV 0.05/10 s, 95% CI − 7.31 to − 5.34; *p* < 0.001) (Fig. 3).

**Table 2:** Hardness test measurements for each type of treated specimen and mean difference from the reference specimen (results of t-tests)

Local adjuvant	Mean HV (HV 0.05/10 s)	Mean difference (HV 0.05/10 s)	95% CI	*p* value
Reference (A)	21.31 ± 1.11	–	–	–
Electrocautery (B)	21.62 ± 1.61	0.310	−1.07 to 1.69	0.640
Phenol (C)	16.41 ± 0.89	−4.900	−5.90 to −3.89	**< 0.001**
Argon beam coagulation (D)	21.12 ± 0.86	−0.190	−1.18 to 0.80	0.690
Liquid nitrogen (E)	14.98 ± 0.84	−6.330	−7.31 to −5.34	< **0.001**

*CI* confidence interval

### Histological Examination of the Depth of Bone Necrosis

**Fig. 4:**
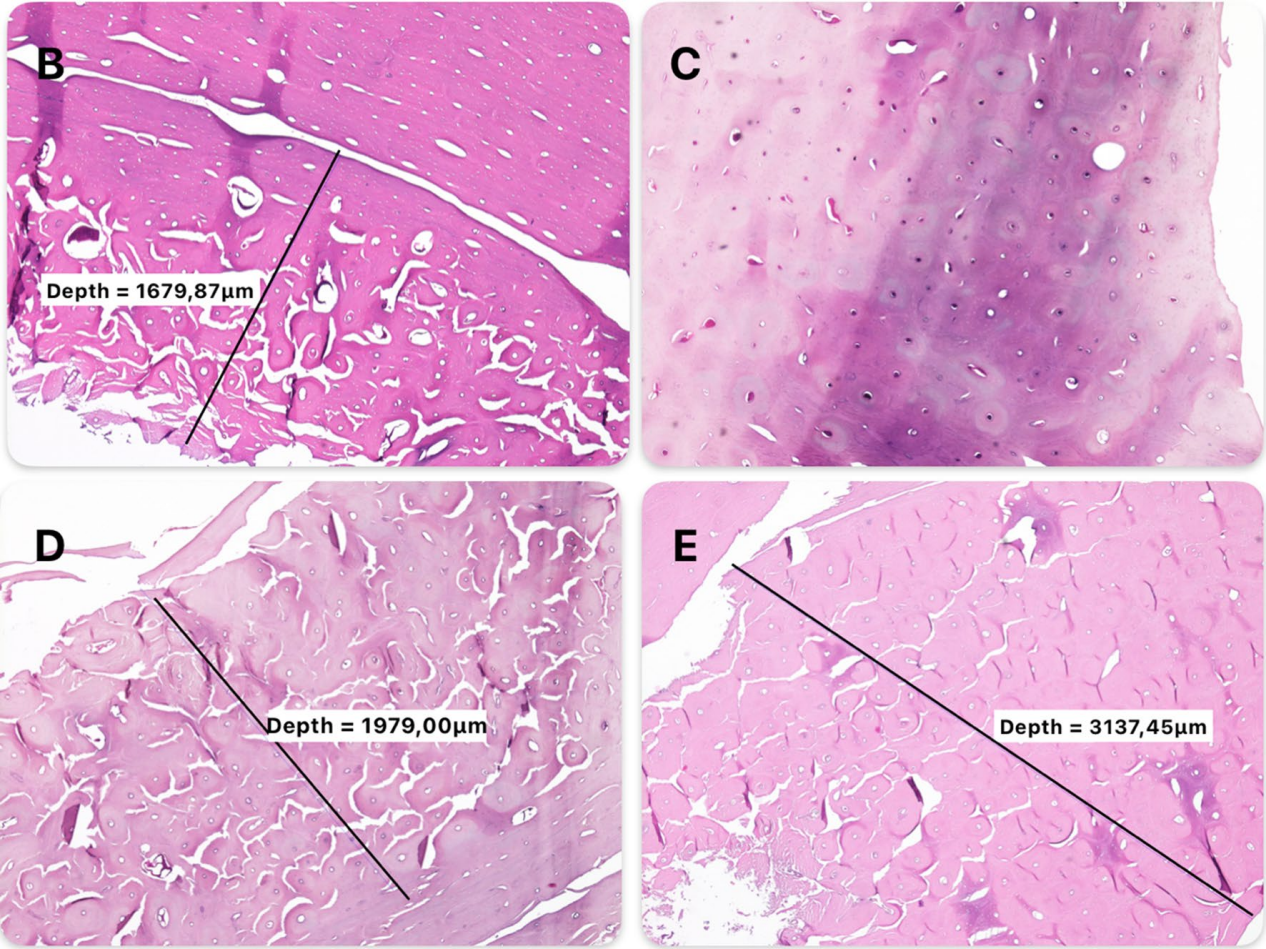
Microscopic hematoxylin and eosin representative slides demonstrating the induced depth of necrosis in porcine cortical bone after local adjuvant application. Types of local adjuvant specimens: **B** electrocautery; **C** phenol; **D** argon beam coagulation; and **E** liquid nitrogen

In the reference specimen, necrosis was not registered on the medular surface of the cortical bone. Thus, the measured mean depth of necrosis corresponded to the mean difference from the reference sample. The mean depth of bone necrosis induced by the thermal local adjuvants was similar; the specimen treated with ABC had a mean value of 1.979 ± 0.89 mm and electrocautery 1.679 ± 0.42 mm. A greater induced depth of necrosis was registered in the specimen treated with liquid nitrogen (3.137 ± 1.9 mm), while the specimen treated with phenolization (0.289 ± 0.12 mm) had the least depth of necrosis (Fig. 4).

## Discussion

Local adjuvants are a beneficial addition to curettage in local recurrence prevention, although their use is related to substantial damage to the surrounding healthy bone tissue.^27^ Studies have shown that the addition of local adjuvants following curettage decreases local recurrence rates, playing a crucial role in reducing the percentage of local recurrences associated with surgical treatment. Local adjuvants focus on eradicating tumors by destroying residual tumor cells, creating a barrier against new growth into surrounding bone tissue. However, there is an absence of consensus on the optimal treatment modality and limited knowledge about the subsequent damage to healthy bone, leading orthopedic surgeons to rely on their experience. The present paper examined the multimodal effect of these chemical and thermal local adjuvants on porcine cortical bone by using micro-CT along with histological and mechanical examination.

To determine the BMD changes in the anorganic tissue of healthy cortical bone after the use of local adjuvants, a micro-CT examination was performed. The present study is the first to examine the changes in BMD among adjuvant therapies for the treatment of bone tumors using micro-CT. As evidenced by the statistical test results (Table 1), the null hypothesis can be rejected for the BMD; therefore, significant differences in density are observed between the reference sample and the samples treated with a local adjuvant. The most noticeable change is observed in the sample treated with the ABC (BMD reduction by 9.2%); institutional studies recorded a high rate of fractures after ABC application and suggested that ABC may lead to an increased risk of postoperative fracture.^29,38^ On the other hand, a retrospective study of 40 patients recorded no postoperative complications.^25^ Our results indicate that the mineral structure of the cortical bone is affected by the local adjuvant; however, the effect evaluated by means of the mean BMD is only in the range of single-digit percentage values. Nonetheless, further examination of the effect of ABC on the mineral structure of the bone is suggested.

The Vickers indentation test is a helpful tool for evaluating the mechanical properties of bone.^35^ One factor that affects bone hardness is the degree of mineralization, and another is collagen, the principal organic matrix in bone.^39^ By examining bone hardness, we determined the effect of local adjuvants on the organic and inorganic matrices of the bone. In a comparative cohort study, van der Heijden et al. compared patients treated with phenol and liquid nitrogen. Despite the similar recurrence rate in both groups, patients treated with liquid nitrogen presented a higher risk of complications, including pathological fractures.^27^ Other studies recorded a decrease in the local recurrence rate when phenol was applied.^19,23^ van der Geest et al. described a 14% risk of postoperative fracture after cryotherapy using liquid nitrogen,^40^ while a lower postoperative fracture rate, using liquid nitrogen, was recorded by Marcove et al.^41^ Our study recorded a notable decrease in bone hardness in the specimens treated with phenol, and even more in those treated with liquid nitrogen. This decrease in bone hardness in the case of phenolization could be explained by protein denaturation, including that of collagen, as well as cell destruction.^42^ The mechanisms of bone damage induced by liquid nitrogen have been examined in detail.^43–45^ In cases of electrocautery and ABC, the null hypothesis cannot be rejected as there is insufficient evidence to assert a significant impact on hardness.

A common method to examine bone damage is histological measurement of the depth of necrosis.^28,46,47^ Previous studies showed a depth of bone necrosis of between 1 and 2.9 mm in specimens treated with ABC, and 0.92 and 3 mm using electrocautery.^24,28,46^ These findings are similar to our measured values. In a recent study, liquid nitrogen was described to induce a greater depth of necrosis, with a wide variance of values (2.54 ± 1.55 mm).^28^ In our examination, slightly higher values were measured (3.137 ± 1.9 mm). However, it is challenging to objectively determine the exposure of the specimens to liquid nitrogen, and this is likely the reason for the wide variance of the measured values. As expected, the least depth of necrosis was measured in the specimen treated with phenol. This could be explained by the limited bone tissue penetration of the phenol.^42^ Similar values of phenolization-induced necrosis depth have been described in the literature.^28,42^

The present study has several limitations. First, porcine femoral bone differs from human bone tissue and cadaverous bone might therefore be preferable. Second, the current study did not include combinations of different local adjuvants and this could be a subject of future research. Third, although high-speed burring is a common component of the operative technique, to reduce bias, it was not used. The strengths of this study include standardized exposure to local adjuvants following the operative technique described in the literature. All specimens were derived from the mid part of the diaphysis of porcine femurs of similar size, and handled and stored identically. Finally, to perform a complete examination of the effect of local adjuvants, we measured the bone density, bone hardness, and depth of necrosis.

## Conclusion

The present study determined the effect of local adjuvants on cortical bone by using micro-CT along with histological and mechanical examination. Considering the limitations, this study was the first attempt to compare the induced changes in terms of bone density and bone hardness. Phenolization and liquid nitrogen application caused a decrease in bone hardness, while ABC and electrocautery showed sufficient penetration without negatively affecting the hardness of the specimens. The bone density of the cortical bone was affected by the local adjuvants, and ABC caused a noticeable change. Liquid nitrogen was described to induce extensive depth of necrosis with a wide variance of values. Conversely, phenol showed limited penetration to the cortical bone.

## Data Availability

The data that support the findings of this study are available from the corresponding author upon reasonable request.
